# Apicomplexan haemoparasites in domestic cats in Romania

**DOI:** 10.1186/s13071-023-05683-7

**Published:** 2023-02-06

**Authors:** Luciana Cătălina Panait, Angela Monica Ionică, Cristina Daniela Cazan, Mircea Coroian, Ana Maria Diacu, Ana Maria Boncea, Cosmin Mateescu, Andrei Daniel Mihalca

**Affiliations:** 1 Department of Parasitology and Parasitic Diseases, Faculty of Veterinary Medicine, University of Agricultural Sciences and Veterinary Medicine of Cluj-Napoca, Calea Mănăștur 3-5, 400372 Cluj-Napoca, Romania; 2 CDS‑9: Molecular Biology and Veterinary Parasitology Unit, Faculty of Veterinary Medicine, University of Agricultural Sciences and Veterinary Medicine of Cluj-Napoca, 400372 Cluj-Napoca, Romania; 3 Microbiology Laboratory, Clinical Hospital of Infectious Diseases of Cluj-Napoca, 23 Iuliu Moldovan, 400348 Cluj-Napoca, Romania; 4 CSV Lunca Bradului, 547380 Mureș, Romania; 5 Falcon Vet Veterinary Private Clinic, 21919 Bucharest, Romania; 6 Agervet Targoviște Veterinary Private Clinic, 130120 Dâmbovița, Romania; 7 Parasitology Consultancy Group, 407056 Corușu, Cluj Romania

**Keywords:** Apicomplexa, Domestic cats, PCR, Romania, Vector-borne pathogens

## Abstract

**Background:**

Apicomplexan haemoparasites are protozoans that infect a variety of domestic and wild animal species, as well as humans. Data regarding haemoprotozoans in domestic cats are limited; therefore, the aim of this study was to assess the occurrence of *Babesia* spp., *Cytauxzoon* spp., and *Hepatozoon* spp. in domestic cats in Romania using molecular tools.

**Methods:**

Blood samples from 371 domestic cats were screened for the presence of piroplasmids. All samples that yielded a visible band in agarose gels were subsequently tested by specific assays targeting the *18S rDNA* of *Babesia* spp., *Cytauxzoon* spp., and *Hepatozoon* spp. Moreover, nested PCR assays targeting mitochondrial genes of *Babesia* spp. were used for screening of all *Babesia* spp. *18S rDNA*-positive samples.

**Results:**

From the total number of sampled cats, 19.4% were positive in the PCR assay targeting piroplasmids. *Babesia* spp. were identified in 15.1% of cats, while 0.5% were positive for *Hepatozoon* spp. Molecular analyses confirmed the presence of *Babesia canis*. No samples were positive for *Cytauxzoon* spp.

**Conclusions:**

The high infection rates of domestic cats with *Babesia* spp. and the need for species differentiation highlight the importance of mitochondrial genes as targets for molecular protocols.

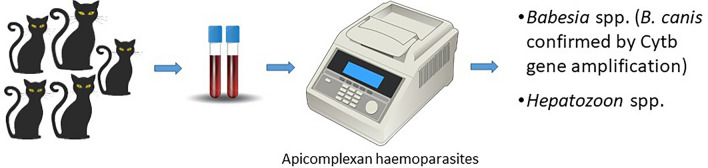

## Background

Apicomplexan haemoparasites are protozoans that infect a wide variety of domestic and wild animals, as well as humans [1, 2]. The complex interactions between domestic animals, wild reservoirs and arthropod vectors favour tick-borne pathogen transmission and increase their geographical distribution [3]. During the last decades, several studies have focused on the detection and characterization of haemoprotozoans in domestic dog populations worldwide [4–7]. However, in domestic cats, data regarding the presence of haemoprotozoans are scarce.

Genus *Babesia* includes more than 100 species, with intraerythrocytic localization in the vertebrate host, causing mild to severe haemolytic diseases [2, 8]. Several ixodid ticks are thought to be involved in their transmission, although vector competence has not been confirmed in all cases [9]. Feline babesiosis is a relatively newly recognized clinical entity, with most available studies originating from South Africa [10]. Non-specific clinical signs such as anaemia, lethargy, and anorexia are described, whereas icterus and fever are inconsistently found [11]. While several *Babesia* spp. have been documented in domestic cats, the species most commonly associated with clinical cases is *Babesia felis* [10]. In Europe, *Babesia microti* [12, 13] and dog-related species such as *Babesia canis* [14, 15], *Babesia vogeli* [15, 16], and *Babesia vulpes* (formerly known as *Theileria annae*) [14, 17] have been detected in domestic cats. Nevertheless, the European cases were rarely associated with clinical manifestations. Recently, *Babesia pisicii* was described in European wild cats, *Felis silvestris* in Romania [18], but its presence in domestic cats has not been documented yet.

*Hepatozoon* spp. are haemogregarines with a life-cycle shared between a wide range of vertebrates as intermediate hosts and various haematophagous arthropods as definitive hosts [19]. The main transmission pathway is represented by the ingestion of the arthropod definitive host containing mature sporozoites by the intermediate host [20]. In domestic cats, *Hepatozoon* spp. were reported for the first time in India [21]. Since then, *Hepatozoon* infections have been found in domestic cats and various wild felids worldwide [22]. In Europe, *Hepatozoon felis* is recognized as the main agent infecting domestic and wild felines [15, 16, 23–36]. *Hepatozoon canis* has also been reported in domestic cats in Europe [28, 29, 37–39]. Recently, a novel species, *Hepatozoon silvestris*, was described in European wild cats from Bosnia and Herzegovina [30] and was further reported in domestic [29, 35, 40] and wild felids [31] in Europe. Feline hepatozoonosis is mostly subclinical, with no significant inflammatory response in association with the presence of meronts in muscle tissue [26, 31].

Feline cytauxzoonosis, first described in the 1970s [41], is a tick-borne disease affecting both domestic and wild felids [42]. Five *Cytauxzoon* species have been described so far in felids *Cytauxzoon felis*, *Cytauxzoon manul*, *Cytauxzoon europaeus*, *Cytauxzoon banethi*, and *Cytauxzoon otrantorum*. *Cytauxzoon felis* is considered endemic to North America, causing a highly fatal disease in domestic cats, in both natural and experimental infections [43]. Bobcats *(Lynx rufus*) are the natural reservoirs [3]. In 2005, *C. manul* was described from Pallas’ cats (*Otocolobus manul*) imported from Mongolia to the USA [44, 45]. In Europe, unnamed isolates of *Cytauxzoon* have been documented in the past decade in domestic cats [28, 35, 39, 46–53], Iberian lynx (*Lynx pardinus*) [54–59], Eurasian Lynx (*Lynx lynx*) [60], and European wild cats [31, 60–62]. In a breeding centre in Russia, *Cytauxzoon* spp. were identified in a serval, a bobcat, seven Amur wild cats, and two domestic cats [63]. Recently, three *Cytauxzoon* species were described in European wild cats: *C. europaeus*, which was identified in several central, eastern, and southern European countries [64–67], and *C. banethi* and *C. otrantorum*, which to date had been identified only in Romania [64]. Additionally, *C. europaeus* was identified in domestic and stray cats in Switzerland [65]. However, no data are available on the clinical significance of these species for domestic cats.

No previous studies are available on apicomplexan haemoparasites in domestic cats in Romania, and data from Eastern Europe is generally very limited. Therefore, the aim of this study was to investigate the occurrence of *Babesia* spp., *Cytauxzoon* spp., and *Hepatozoon* spp. in domestic cats in Romania using highly specific polymerase chain reaction (PCR) protocols and to identify the potential risk factors associated with these infections.

## Methods

### Sample and data collection

Blood samples from 371 domestic cats were collected between October 2017 and May 2019. The animals included in the study were client-owned (referred to urban private veterinary clinics or from rural areas), stray (from animal shelters), or feral (living in cat colonies). Whole blood samples were collected into sterile tubes containing anticoagulant (ethylenediaminetetraacetic acid [EDTA] or citrate) after obtaining informed consent for patient enrolment from the owners. The samples were stored at −20 °C until further analysis.

Outdoor access and age (cats older than 4 months) were considered as inclusion criteria. When available, epidemiological data (sex, age, breed, lifestyle, habitat, and ecoregion) were noted for each animal.

### Molecular and phylogenetic analyses

Genomic DNA was isolated using the Isolate II Genomic DNA Kit (Meridian Bioscience, London, UK) from 200 µl of whole blood, following the manufacturer’s instructions. Each DNA sample was stored at −20 °C until further use.

A highly sensitive nested PCR protocol targeting a 561–613-base pair (bp) fragment of 18S ribosomal DNA (*18S* *rDNA*) of piroplasmids (*Cytauxzoon* spp., *Babesia* spp., *Theileria* spp., and *Hepatozoon* spp.) was used for initial screening. Due to the high number of weak bands and the low quality of the sequences obtained, all positive or dubious samples were subsequently screened by specific nested PCR assays targeting the *18S rDNA* of *Babesia* spp., *Cytauxzoon* spp., and *Hepatozoon* spp. Moreover, nested PCR assays targeting the cytochrome *b* (*Cytb)* and cytochrome *c* oxidase subunit I (*COI)* genes of *Babesia* spp. were used for screening of all *Babesia* spp.-positive samples. Primers and PCR conditions are detailed in Table 1.

**Table 1:** Primer pairs and PCR conditions used for PCR amplification

Target and genetic marker	Primer name and sequence (5′–3′)	Annealing temperature/amplicon length	Reference
Piroplasmida *18S rDNA*	BTH_F: CCTGMGARACGGCTACCACATCT	60 °C/686–747 bp	[91]
	BTH_R: TTGCGACCATACTCCCCCCA		
	GF2: GTCTTGTAATTGGAATGATGG	50 °C/561–613 bp	[92, 93]
	GR2: CCAAAGACTTTGATTTCTCTC		
*Babesia* spp. *18S rDNA*	Bc_F1: CGTAGTTGTATTTTTGCGT	50 °C/≈ 430 bp	[94]
	GR2: CCAAAGACTTTGATTTCTCTC		
	Bc_F2: CATTTGGTTGGTTATTTCGTTTT	53 °C/376 bp	
	Bc_R1: GTTCCTGAAGGGGTCAAAAA		
*Babesia* spp. *Cytb*	Bc_cytb_F1: TGGTCWTGGTATTCWGGAATG	50 °C/≈ 700 bp	[95]
	Bc_cytb_R1: AAGMYARTCTYCCTAAACATCC		
	Bc_cytb_F2: RATKAGYTAYTGGGGAGC	48 °C/≈ 580 bp	
	Bc_cytb_R2: GCTGGWATCATWGGTATAC		
*Babesia* spp. *COI*	Bab_For1: ATWGGATTYTATATGAGTAT	45 °C/1250 bp	[95]
	Bab_Rev1: ATAATCWGGWATYCTCCTTGG		
	Bab_For2: TCTCTWCATGGWTTAATTATGATAT	49 °C/980 bp	
	Bab_Rev2: TAGCTCCAATTGAHARWACAAAGTG		
*Cytauxzoon* spp. *18S rDNA*	7549F: GTCAGGATCCTGGGTTGATCCTGCCAG	60 °C/1726 bp	[64]
	7548R: GACTGAATTCGACTTCTCCTTCCTTTAAG		
	Cyt-SSU-F2: CATGGATAACCGTGCTAATTG	53 °C/1335 bp	
	Cyt-SSU-R4: AGGATGAACTCGATGAATGCA		
*Hepatozoon* spp. *18S rDNA*	HAM1F: GCCAGTAGTCATATGCTTGTC	52 °C/≈ 1700 bp	[95]
	HPF2R: GACTTCTCCTTCGTCTAAG		
	EF-M: AAAACTGCAAATGG CTCATT	55 °C/≈ 1600 bp	
	Hep1615R: AAAGGGCAGGGACGTAATC		

First-round reactions were carried out in a total volume of 15 μl containing 7.5 μl of 2× PCRBIO Taq Mix Red (PCR Biosystems, London, UK), 400 nM of each primer, and 1 μl of template DNA, except for the amplification of the partial *18S rDNA* of piroplasmids that was performed using 2 μl of DNA. Amplification of the second round was carried out in a 25 μl reaction mixture consisting of 12.5 μl of 2× PCRBIO Taq Mix Red (PCR Biosystems, London, UK), 400 nM of each primer, and 1 μl of the first PCR round as the template. One positive control, consisting of DNA from carnivores previously confirmed as positive for the targeted pathogens [18, 64, 68], and a negative control represented by sterile water were included in each reaction set. Amplicons were visualized on 1.5% agarose gels stained with ECO Safe Nucleic Acid Staining Solution (PacificImage Electronics, New Taipei City, Taiwan).

Products of expected size were cut from gels and purified using the Gel/PCR DNA Fragments Extraction Kit (Geneaid Biotech Ltd., New Taipei City, Taiwan). PCR products were sequenced with Sanger sequencing technology in both directions (performed at Macrogen Europe, Amsterdam, the Netherlands) using the amplification primers. Chromatograms were assembled and edited using Geneious 4.8.5 software [69], and consensus sequences were compared to homologous sequences available in the GenBank^®^ database using the NCBI Basic Local Alignment Search Tool (BLASTn) analysis. Protein coding gene sequences were translated to corresponding amino acids, based on the protozoan mitochondrial genetic code, to guide nucleotide alignment.

To investigate the relations among *Babesia* spp., phylogenetic analysis of the *18S rDNA* and *Cytb* genes was performed using MEGA X software [70] based on all unique sequences obtained in the present study and available sequences of *Babesia* sensu stricto clade VI, according to Schnittger et al. [2], longer than 300 bp. In the case of *Cytb*, *B. pisicii* was also included in the analysis. Two *18S rDNA* sequences of *H. felis* and two *Cytb* sequences of *Theileria parva* were used as outgroups. In the case of both datasets, the sequences were aligned using the ClustalW algorithm, resulting in a final alignment of 47 sequences for the *18S rDNA* and 25 for the *Cytb* gene, respectively. The phylogenetic trees were inferred by the maximum likelihood method. The best-fit substitution models, with the lowest Bayesian information criterion (BIC) scores, as calculated by the software, were used: Kimura 2-parameter, using a discrete gamma distribution (K2+G) for the *18S rDNA*, and Tamura 3-parameter, using a discrete gamma distribution (T92+G) for the *Cytb* gene, respectively. Branch support was estimated using 1000 bootstrap replicates. The resulting tree topologies were visualized and edited in FigTree v1.4.4 and Inkscape 0.94.

### Statistical analyses

Statistical analyses were performed using R software v. 4.0.5 (R Foundation for Statistical Computing, Vienna, Austria). The prevalence and its 95% confidence interval (CI), overall and differentiated by different epidemiological data, were calculated, and the existence of a statistical association between PCR positivity rates and explanatory variables (sex, age, breed, lifestyle, habitat, and ecoregion) was evaluated by Fisher’s exact test. *P*-values less than 0.05 were considered statistically significant.

## Results

From the total number of sampled cats, 72 (19.4%, 95% CI 15.4–23.4) showed a visible band in the PCR targeting the *18S rDNA* of piroplasmida. From these, 56 samples (15.1%, 95% CI 11.5–18.7) yielded an amplicon in the assay targeting the *18S rDNA* of *Babesia* spp. The sequences represented five unique haplotypes. The most common haplotype (BHF014) was detected in 52 samples. The remaining four haplotypes were represented by one sample each, differing from the main haplotype by three single-nucleotide polymorphism (SNP) sites and one indel (1.12%; 4/356 nucleotides [nt]), in the case of the sample represented by ARF008, and one SNP site in the case of the other three haplotypes, respectively. The BLASTn analysis of the obtained haplotypes showed 98.9–100% nucleotide sequence identity with *B. canis* from dogs from Lithuania (GenBank accession numbers: MN078319-MN078323), Iran (MN173223), or Bosnia and Herzegovina (MK107800-MK107806). All sequences represented by unique haplotypes were deposited in GenBank (accession numbers OL342311-OL342315).

The amplification of the *Cytb* gene of *Babesia* spp. was successful in six samples, while no positivity was noticed in the assay targeting the *Babesia* spp. *COI* gene. Two haplotypes with 99.8% identity were detected in the six samples (1 SNP/477 nt). The sequences displayed a 99.6–99.8% identity to *B. canis* from the USA (KC207822) or *B. canis* reported in a dog from Poland (MK024727) and 99.4–99.6% identity to *B. canis* detected in European wild cats in Romania (MW938761). The two sequences were deposited in the GenBank database under the accession numbers OL355016 and OL355017.

From the 72 samples positive in the initial screening, two yielded an amplicon in the assays targeting the 1600-bp fragment of the *18S rDNA* of *Hepatozoon* spp., resulting in an overall prevalence of 0.5% (95% CI 0.0–1.3). However, direct sequencing resulted in four short sequences (two forward and two reverse), that showed an overall identity of 99–100% to *Hepatozoon* sp. identified in ocelots from Brazil (KX776299, KX776303) or reptiles from Spain (MG787243), or *H. felis* reported in lions from India (KX01729, ON075470) or wild cats from Hungary (OM422756). The two samples were also positive for *Babesia* sp. The remaining 16 samples tested negative in all specific protocols. All sampled domestic cats were PCR negative for *Cytauxzoon* spp.

Statistical analyses showed that the *Babesia* spp. infection rate was statistically higher in cats from the Pannonian ecoregion than in the rest of the country, at a *P*-value of 0.03. No associations were found between the presence of the pathogens and other categorical variables (Table 2).

**Table 2:** Prevalence of *B. canis* and *Hepatozoon* spp. in association with epidemiological data

	Total (%^a^)	Positive samples (%, 95% CI)	
		*B. canis*	*Hepatozoon* spp.
Sex			
Males	191 (51.4)	28 (14.7, 95% CI 9.6–19.7)	2 (1.0, 95% CI 0.0–2.5)
Females	172 (46.4)	28 (16.3, 95% CI 10.8–21.8)	0
Not available	8 (2.2)	0	0
Age			
< 3 years old	178 (48.0)	28 (15.7, 95% CI 10.4–21.1)	0
≥ 3 years old	171 (46.1)	24 (14.0, 95% CI 8.8–19.2)	2 (1.2, 95% CI 0.0–2.8)
Not available	22 (5.9)	4 (18.2, 95% CI 2.0–34.3)	0
Breed			
European shorthair	338 (91.1)	50 (14.8, 95% CI 11.0–18.6)	2 (0.6, 95% CI 0.0–1.4)
Other breeds	33 (8.9)	6 (18.2, 95% CI 5.0–31.3)	0
Lifestyle			
Client-owned	314 (84.6)	49 (15.6, 95% CI 11.6–19.6)	2 (0.6, 95% CI 0.0–1.5)
Stray	30 (8.1)	2 (6.7, 95% CI 0.0–15.6)	0
Feral	27 (7.3)	5 (18.5, 95% CI 3.9–33.2)	0
Habitat			
Urban	272 (73.3)	43 (15.8, 95% CI 11.5–20.1)	1 (0.4, 95% CI 0.0–1.1)
Rural	99 (26.7)	13 (13.1, 95% CI 6.5–19.8)	1 (1.0, 95% CI 0.0–3.0)
Ecoregion			
Continental	218 (58.8)	25 (11.5, 95% CI 7.2–15.7)	2 (0.9, 95% CI 0.0–2.2)
Steppe	10 (2.7)	0	0
Alpine	36 (9.7)	8 (22.2, 95% CI 8.6–35.8)	0
Pannonian	107 (28.8)	23 (21.5, 95% CI 13.7–29.3)^b^	0

^a^The percentages were computed from the total number of animals included in the study (*n* = 371)

^b^Statistically significant relationship (*P* = 0.03)

**Fig. 1:**
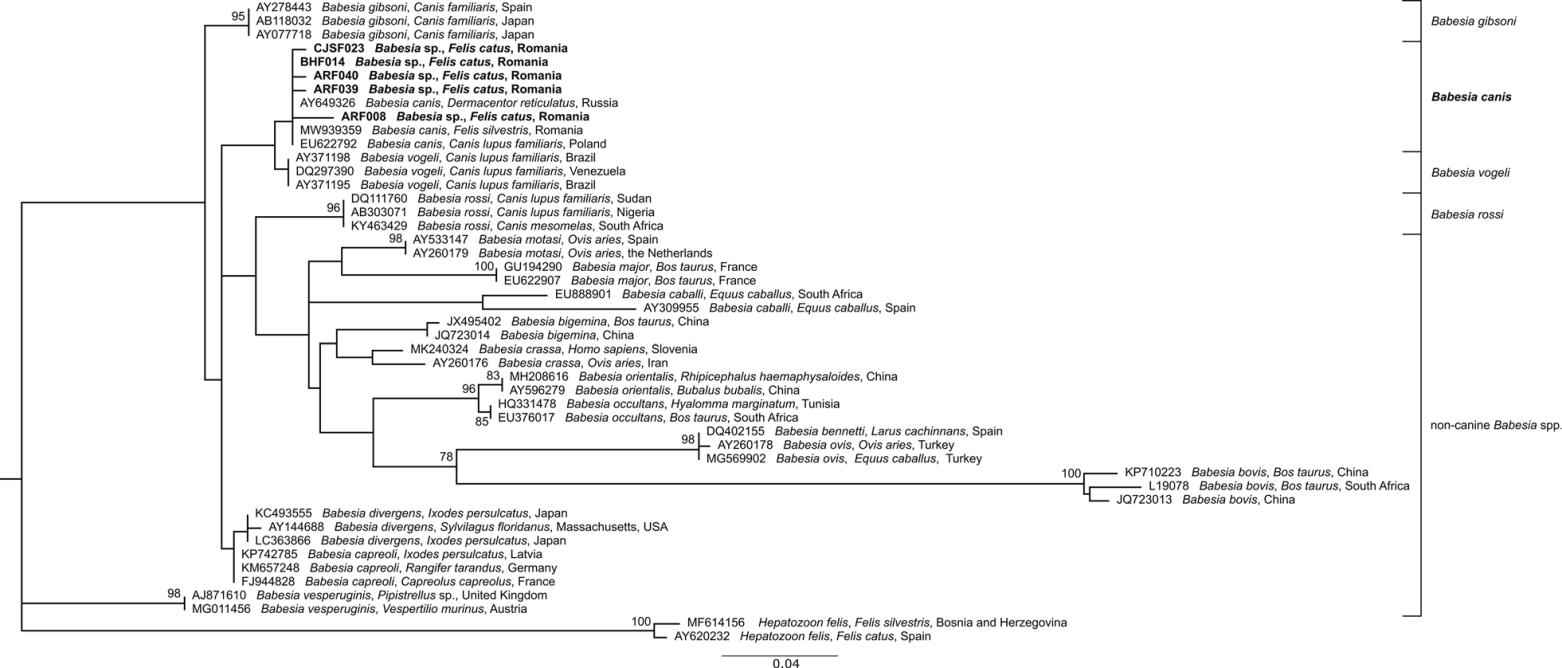
Maximum likelihood tree based on partial *18S rDNA* sequences obtained in the current study (in bold) and sequences of *Babesia* sensu stricto species (clade VI sensu Schnittger et al. [2]). Only bootstrap values above 75% are displayed. The scale bar indicates the number of nucleotide substitutions per site. The GenBank accession number, assigned name, host, and country of origin are indicated for each sequence, if available

**Fig. 2:**
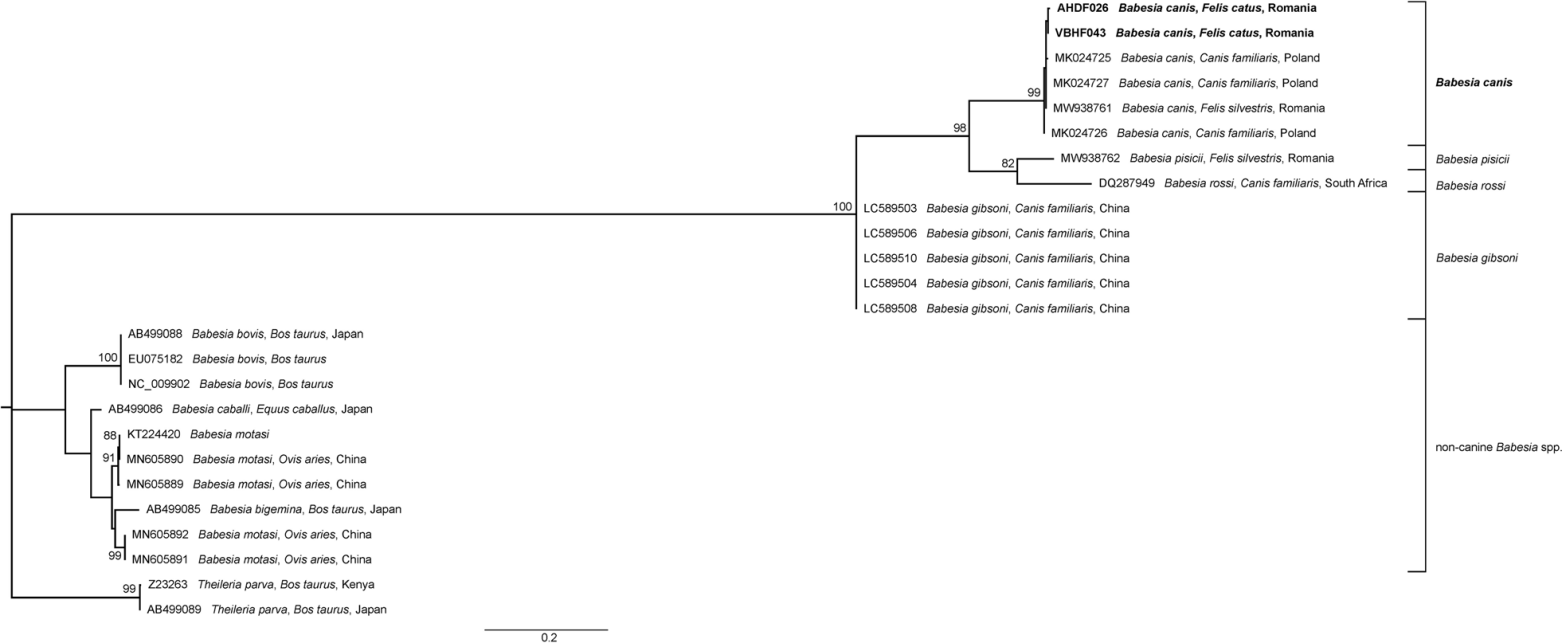
Maximum likelihood tree based on *Cytb* sequences obtained in the current study (highlighted in bold) and sequences of *Babesia* sensu stricto species (clade VI sensu Schnittger et al. [2]). Only bootstrap values above 75% are displayed. The scale bar indicates the number of nucleotide substitutions per site. The GenBank accession number, assigned name, host, and country of origin are indicated for each sequence, if available

Phylogenetic analyses of both *18S rDNA* (Fig. 1) and *Cytb* (Fig. 2) sequences confirmed the affiliation of our sequences to the *B. canis* clade, clustering together with other *B. canis* sequences from dogs, European wild cats or ticks, in a sister clade to *B. vogeli*, in the case of *18S rDNA*, and to *B. pisicii* and *Babesia rossi* (bootstrap value: 98), in the case of *Cytb*.

## Discussion

The results of the current study confirmed for the first time that *B. canis* and *Hepatozoon* spp. are circulating in domestic cats in Romania. The *18S rDNA*, a highly conserved region, is the primary PCR target used in studies addressing the diagnosis of piroplasmids [71, 72]. However, several studies have questioned its ability to differentiate between closely related species [2, 18, 64, 73, 74]. In the present study, BLAST analyses of the *18S rDNA* sequences showed 99–100% identity with different sequences of *B. canis*, although the presence of this species was confirmed in only six samples by *Cytb* gene amplification and analyses. *Babesia canis* was previously reported in domestic cats in Europe, the data being supported exclusively by relatively short *18S rDNA* fragments [14, 15]. Therefore, as previously highlighted [18], our recommendation remains to avoid using protocols targeting the *18S rDNA* for piroplasmid species differentiation, and samples amplified by these protocols should be considered as *Babesia* sp. PCR protocols based on mitochondrial gene detection can be successfully used for species confirmation, but have shown lower sensitivity than *18S rDNA* amplification protocols (100 to 1000 times lower sensitivity than the protocol targeting the 376-bp fragment of *B. canis* *18S rDNA*) [18, 74].

Several *Babesia* species have been documented in domestic cats worldwide: *B. felis* [10], *B. leo* [75], *B. lengau* [76], and *Babesia* sp. cat Western Cape [75] in Africa; *B. hongkongensis* [77], *B. canis presentii* [78], *B. panickeri* [79], and *B. vogeli* [80, 81] in Asia; and *B. vogeli* and *B. gibsoni* in the Americas [82]. In Europe, epidemiological data on *Babesia* infection in domestic cats are limited to a few molecular studies targeting the *18S rDNA*, with *B. canis* reported in Spain and Portugal [14, 15], *B. vogeli* in Portugal [15, 16], and *B. microti* in Italy [12, 13]. *Babesia canis* was also recently reported in wild cats from Romania [18].

Traditionally, the naming of *Babesia* spp. has been based on their assumed host specificity and morphological characters [2]. However, as already discussed by others [18, 83], the presence of *B. canis* DNA was detected in several other non-canid hosts, such as bats [84] and horses [85]. Furthermore, *B. canis* was molecularly detected in mice experimentally fed *Dermacentor reticulatus*-positive ticks, raising the possibility of oral transmission through vectors [83]. This hypothesis was also put forward by Hornok et al. [86] when *B. canis* DNA was identified in insectivorous bat faeces. Cats can ingest ticks, either with their prey (immature *D. reticulatus* feed on micromammals such as mice and voles [87]) or due to their grooming behaviour.

In wild cats in Romania, a recent study noted a prevalence of *Babesia* spp. infection of 39.2% [18]. However, in this previous study on wild cats [18], *B. canis* was confirmed in only one sample by mitochondrial marker assay, while in three samples, a novel species, *B. pisicii*, was described. The presence of this species was not confirmed in domestic cats.

The geographical distribution of *Hepatozoon* spp. in domestic cats is apparently wide in Europe, with reports originating mainly from Mediterranean countries, such as Spain [23, 24, 28, 37], Portugal [15, 16], Italy [29, 32, 35], Cyprus [27], Greece [36], and France [39], but also from Central Europe: Austria [33] and Switzerland [40]. In our study, the exact identity of the species involved could not be established due to the low quality of the obtained sequences. The low parasitaemia level observed in domestic cats during other studies [25, 35], as well as the predominance of subclinical manifestations in *Hepatozoon* spp. infection [26, 35], most likely contributed to these impediments. Cloning procedures presumably have the ability to improve the molecular results.

In recent decades, *Hepatozoon* infection in felids has been increasingly reported worldwide, usually with low infection rates, but ranging up to 37.9% in Cyprus [27]. The overall prevalence of *Hepatozoon* spp. in the present study was 0.5%, similar to that found in Spain [37] or Italy [32].

 Piroplasms of the genus *Cytauxzoon* have gained increased interest in recent years in Europe, due to the high prevalence observed in wild felids [54–67] and occasional clinical reports [48–51, 53, 65]. Despite the diversity and common occurrence of *Cytauxzoon* infection in European wild cats [64], no positive domestic cat was found in this study. Similar results have been obtained in other large-scale surveys conducted on outdoor, stray, or feral cats in Italy [13, 88, 89] and Greece [90]. The reports of *Cytauxzoon* sp. in domestic cats from Europe either are clinical case reports [48–51, 53] or represent findings in asymptomatic cats from Mediterranean regions: Spain [28, 46], France [39], or Italy [35, 47, 52]. At the moment, no report of *Cytauxzoon* is available from healthy cats in other parts of Europe.

## Conclusions

To the authors’ knowledge, this is the first report of *Babesia* and *Hepatozoon* spp. in domestic cats in Romania. Moreover, the study shows high infection rates with *Babesia* spp. in domestic cat populations, confirms the presence of *B. canis* by using a specific genetic marker, and highlights the importance of using mitochondrial genes as targets for PCR analyses that are aimed at piroplasmid species differentiation. Nevertheless, no *Cytauxzoon* spp.-positive samples were identified. Further studies are required to develop highly sensitive PCR assays targeting the *Cytb* or *COI* gene of *Babesia* spp., and to clarify the clinical implication of this pathogen in domestic cats.

## Data Availability

The datasets supporting the conclusions of this study are included in this published article.
